# Dual impacts of lncRNA XIST and lncRNA SNHG5 on inflammatory reaction and apoptosis of endothelial cells via regulating miR‐155/CARHSP1 axis

**DOI:** 10.1111/jcmm.15940

**Published:** 2020-10-14

**Authors:** Xianjing Song, Chuang Yang, Jing Chang, Xin Xue

**Affiliations:** ^1^ Department of Cardiology The Second Hospital of Jilin University Changchun City China; ^2^ Clinical Laboratory The Second Hospital of Jilin University Changchun City China

**Keywords:** apoptosis, atherosclerosis, inflammatory cytokines, LncRNA CARHSP1, LncRNA SNHG5, LncRNA XIST, miR‐155, ox‐LDL

## Abstract

Considering the significance of lncRNA/miRNA axis in explaining atherosclerosis (AS) progression, this investigation was intended to clarify whether lncRNAs XIST/SNHG5 would regulate AS aetiology by sponging miR‐155, an AS‐promoting molecule. We altogether recruited 367 patients who were examined by coronary angiography, and meanwhile, human coronary artery endothelial cells (HCAECs) were purchased to establish cells models via ox‐LDL treatment. The study results indicated that lowly expressed XIST/SNHG5 and highly expressed miR‐155 were frequently detectable among AS patients who showed severe stenosis and possessed high triglyceride (TG), low‐density lipoprotein cholesterol (LDL‐C) and high‐sensitivity C‐reactive protein (hs‐CRP) levels. Besides, HCAECs treated by ox‐LDL released large amounts of inflammatory cytokines, and their apoptosis rate was also raised. Moreover, expressions of XIST and SNHG5 declined markedly within ox‐LDL‐treated HCAECs, whereas miR‐155 expression significantly ascended. Transfection of pcDNA‐XIST and pcDNA‐SNHG5 both reduced the expression of TNF‐α, IL‐6, IL‐8 and IL‐1β within HCAECs and also dampened the apoptotic tendency of HCAECs. Co‐treatment of pcDNA‐XIST and pcDNA‐SNHG5 produced a larger effect on HCAEC activity than pcDNA‐XIST or pcDNA‐SNHG5 alone. Furthermore, miR‐155, modified by XIST and SNHG5, was capable of reversing the impacts of XIST and SNHG5 on HCAEC activity. Eventually, CARHSP1 was activated by XIST and SNHG5, and its overexpression dwindled impacts of miR‐155 mimic on proliferation and inflammation response of HCAECs. In conclusion, targeting XIST and SNHG5 might be an ideal alternative in delaying AS progression, allowing for their repression of downstream miR‐155.

## 1 INTRODUCTION

Atherosclerosis (AS), a common disorder that seriously endangers human health, is considered as the chief pathological basis of ischaemic cardiovascular and cerebrovascular disorders, such as coronary heart disease, cerebrovascular disease and thromboembolic disease.^1^ The mortality triggered by cardiovascular diseases was estimated to achieve 36% of global death by 2020,^2^ which stressed the urgency of figuring out corresponding treatments. Mechanically, AS, initiated by malfunction of endothelial cells (ECs), was documented to result from disorders in lipid metabolism and chronic inflammation.^3^ More specifically, in the wake of oxidative stress‐forced endothelial dysfunction, endothelial permeability changed, and mononuclear macrophages began to aggregate and release inflammatory factors, which ultimately intensified proliferation of vascular smooth muscle cells and prompted development of AS.^4^ Hence, capturing the aetiology that explained proper functioning of ECs appeared pivotal to alleviate AS symptoms.

A wide variety of investigations have emphasized the participation of miRNAs, especially miR‐155, in modulating function of ECs.^5^ For instance, miR‐155 was competent in obstructing EC proliferation and repressing neovascularization by negative modification of angiotensin II type‐1 receptor (AGTR1).^6^, ^7^ Besides, V‐Ets‐erythroblastosis virus E26 oncogene homologue 1 (ETS‐1), also targeted and modified by miR‐155, was indispensable to propelling vascular remodelling and controlling inflammation.^8^ To sum up, miR‐155 mattered in regulating both inflammation and apoptosis of ECs, and its upstream regulator genes might exert profound effects on AS progression owing to their direct control over miR‐155.

As put forward by the competitive endogenous RNA (ceRNA) hypothesis,^9^ lncRNAs could sponge miRNAs and then prevent miRNAs from suppressing the action of their downstream genes. As a matter of fact, the lncRNA/miRNA axes have been documented to involve in regulating endothelial function. In particular, lncRNA LOC100129973 served to promote apoptosis of ECs by targeting miR‐4707‐5p and miR‐4757, which was associated with incremental expression of such apoptins as API5 and BCL2LI2.^10^ Besides, apoptosis of ECs was also encouraged by lincRNA‐p21 sponging miR‐130b, as proved by the luciferase reporter gene assay.^11^ Notably, lncRNA X inactive specific transcript (XIST), with a full length of 17 kb and situated in the long arm of X chromosome, was up‐regulated in oxidized low‐density lipoprotein (ox‐LDL)‐treated ECs,^12^ which implied that XIST might participate in modulating EC function and even AS development. Additionally, there existed a sponged regulation between lncRNA XIST and miR‐155 within breast cancer cells,^13^ which provoked a speculation that lncRNA XIST might be implicated in AS aetiology via its sponging miR‐155. Concerning another lncRNA studied here, the lncRNA SNHG5 was also suggested to negatively regulate miR‐155 expression within melanoma cells,^14^ yet whether this regulation also worked in ECs remained uncertain. Furthermore, the SNHG5 seemed adept at modifying cell proliferation and apoptosis, such as withstanding oxaliplatin‐triggered apoptosis of colorectal cancer cells ^15^ and evoking inordinate proliferation of melanocytes.^16^ Nonetheless, few investigations have been carried out to elucidate the possibility of SNHG5 in regulating activities of ECs.

Taken together, lncRNAs XIST and SNHG5 were potentially capable of disturbing EC function by acting upon miR‐155, and this investigation was intended to clarify whether this regulation actually worked, which might conduce to developing AS treatments in future.

## 2 MATERIALS AND METHODS

### 2.1 Collection of AS patients

Totally 367 AS patients, identified by coronary angiography, were recruited from The Second Hospital of Jilin University since July 2017 until March 2018. The participants were all unrelated individuals, and their displayed > 50% stenosis in the diameter of left main trunk, anterior descending branch, circumflex artery and right coronary artery. Stenosis of their coronary artery lesions was graded by modified Gensini scoring method.^17^ On the other hand, the healthy group was made up of 219 non‐AS individuals who were also confirmed by coronary angiography. Furthermore, participants of this investigation were excluded if they were plagued by severe liver/renal insufficiency, malignant tumour, acute pericarditis, acute myocarditis, pulmonary embolism, congenital heart disease, connective tissue disease, autoimmune disease, peripheral/cerebrovascular disease or acute infection. All the patients have signed informed consents prior to participation, and this project was approved by The Second Hospital of Jilin University and the ethics committee of The Second Hospital of Jilin University.

### 2.2 Cell culture

Human coronary artery endothelial cells (HCAECs) of Sciencell Company were provided by Yuhengfeng Technology Co., Ltd (Beijing, China). The HCAECs were cultured in high‐glucose DMEM that incorporated 10% inactivated foetal bovine serum (FBS), 100 U/mL penicillin and 100 U/ml streptomycin, and they were placed within an incubator of 5% CO_2_ and 37°C. HCAECs cultivated to the logarithmic growth phase were taken for following experiments.

### 2.3 Cell transfection and cell treatment

At the time of > 70% confluence, 5 × 10^4^ HCAECs seeded in 24‐well plates were transfected by si‐XIST, pcDNA3.1‐XIST, si‐SNHG5, pcDNA3.1‐SNHG5, miR‐155 mimic, miR‐155 inhibitor, pcDNA3.1‐calcium‐regulated heat stable protein 1 (CARHSP1) and si‐CARHSP1, according to instructions of Lipo3000 kit (Invitrogen, USA). With respect to ox‐LDL treatment, HCAECs growing to 80%‐90% confluence were held within G0/G1 phase after being starved for 10‐12 hours. Then, the HCAECs were, respectively, treated by 25, 50, 100 and 200 μg/mL ox‐LDL for 24 hours. All these experiments were repeated for ≥ 3 times.

### 2.4 MTT assay

HCAECs grown to monolayer confluence were de‐adhered by addition of 0.25% trypsin (Gibco, USA), and the HCAECs were then diluted into single‐cell suspension with supplementation of 10% FBS‐containing DMEM. HCAECs at the density of 1 × 10^5^/mL were inoculated into 96‐well plates, followed by 24‐h cell culture in 5% CO_2_ at 37°C. Then, 20 μL MTT (5 mg/mL) was added to each well to further cultivate HCAECs for 4 hours. After discarding the culture medium, 150 μL dimethyl sulphoxide (DMSO) was injected into each well. The mixture was gently shaken for 12 minutes to completely dissolve the blue crystals. Finally, each well was measured on a microplate reader to obtain their absorbance value at the wavelength of 570 nm. All these experiments were repeated for ≥ 3 times.

### 2.5 Plate cloning assay

HCAECs of logarithmic growth phase were digested to a concentration of 100 per well, and then they were incubated in 5% CO_2_ at 37°C for 2 weeks. The culture was terminated when colonies appeared macroscopically. After rinsage with water, 500 μL methanol crystal violet solution (0.1%) was added to each well to stain the cells, and then cell colonies were counted. All these experiments were repeated for ≥ 3 times.

### 2.6 Cell apoptosis assay

The digested HCAECs were centrifuged at 1000 r/min for 5 minutes, and then pre‐cooled phosphate buffer solution (PBS) was added to re‐suspend cells at 4°C. The cells were centrifuged again at 1000 r/min for 5 minutes, and the sediments were blended by 400 μL binding buffer (eBioscience, USA) to re‐suspend cells. Subsequently, the cells were mixed by 5 μL Annexin V/FITC (eBioscience, USA), followed by 15‐min incubation in the dark. After that, the cells were treated by 10 μL PI (eBioscience, USA), and the resultant samples were gathered to conduct flow cytometry analysis (model: FACSVerse flow detector, BD Biosciences, USA). All these experiments were repeated for ≥ 3 times.

### 2.7 Reverse transcription‐polymerase chain reaction (RT‐PCR)

Total RNAs, extracted from tissues and HCAECs by addition of TRIzol reagent (Invitrogen, USA), were determined on a Nano‐100 micro spectrophotometer, so as to acquire their purity and concentration. Aided by the RT kit (Invitrogen, USA), RNAs (2 ng) were reversely transcribed into cDNAs, following reactions of 37°C for 5 minutes, 85°C for 5 seconds and 4°C for 10 minutes. The RT reaction system utilized was comprised of 1 μL oligo (dT), 4 μL 5 × reaction buffer, 2 μL Dntp (10 mmol/L), 1 μL RilboLock^TM^ RNase inhibitor (40 U/μL), 1 μL RevertAid^TM^ M‐Mulv Reverse Transcription (200 U/μL). Afterwards, guided by the specification of SYBR Green kit (TaKaRa, Japan), the acquired cDNAs were arranged to perform PCR on a Rotor Gene3000 real‐time PCR instrument. The PCR system was made up of 1 μL cDNAs (500 ng/200 μL), 0.6 μL primer (10 pmol/L), 9 μl 2 × SYBR Green PCR Master Mix and 8.4 μl RNase‐free water. And the reaction condition of PCR was designated as (a) 95°C for 5 minutes, and (b) 40 cycles of 95°C for 5 seconds and 60°C for 34 seconds. Ultimately, expression levels of target genes were calculated according to 2‐^△△Ct^ method. All these experiments were repeated for ≥ 3 times.

### 2.8 Western blotting

The HCAECs digested by 0.25% trypsin were dissociated after addition of RIPA lysate, which included 50 mmol/L Tris‐HCl (pH 7.4), 150 mmol/L NaCl, 1% NP‐40 and 0.1% SDS. The concentration of extracted total proteins was determined by feat of BCA method. Then, 40 μg denatured total protein was arranged to perform 10% SDS‐PAGE, which was then transferred onto PVDF membrane under semi‐dry conditions. After 1‐h blockage at room temperature, primary antibodies (rabbit anti‐human, Abcam, USA) against Bax (1:2000, Catalogue: ab32503), Bcl‐2 (1:1000, Catalogue: ab32124), cleaved caspase‐3 (1:500, Catalogue: ab32042), TNF‐α (1:2000, Catalogue: ab6671), IL‐6 (1:500, Catalogue: ab6672), IL‐8 (1:10, Catalogue: ab7747), IL‐1β (1:100, Catalogue: ab7632), CARHSP1 (1:1000, Catalogue: ab96677) and GAPDH (1:10 000, Catalogue: 181 602) were added to incubate proteins at 4°C for overnight. The membrane, having been rinsed by 1 × TBST for 3 times, was mixed with goat anti‐rabbit IgG (H&L) antibodies (1:3000, Catalogue: ab6721) for another 1‐h incubation at room temperature. Next, luminescent solution was dropped onto the membrane, which was then placed in a chemi‐luminescence imager for exposure and development. The relative expression of proteins was calculated by ImageJ software, with GAPDH as the internal reference. All these experiments were repeated for ≥ 3 times.

### 2.9 Double luciferase reporter gene assay

The miR‐155‐binding sites located within XIST, SNHG5 and CARHSP1 were predicted using StarBase v2.0, and then corresponding sequences of XIST, SNHG5 and CARHSP1 were amplified through RT‐PCR. After undergoing agarose gel electrophoresis (AGE), the PCR products (0.5 pmol/L, 1 μL) were purified via usage of gel extraction kit (TaKaRa, Japan) and were then connected to 0.5 μL pGL3 vector (0.2 pmol/L) (Promega, USA). After that, every 10 μL connection product was mixed by 50 μL DH5α *E. coli* competent cells (TaKaRa, Japan), and penbritin was added to screen drug‐resistance cells. The connection plasmid and empty plasmid were then extracted to experience double‐enzyme (ie *Xho*I and *Hind* III) restriction, and sequences of the products were determined. After all these procedures, pGL3‐XIST‐Wt, pGL3‐SNHG5‐Wt and pGL3‐CARHSP1‐Wt reporter vectors were successfully established. In a similar way, the miR‐155‐binding sites in XIST, SNHG5 and CARHSP1 were mutated, and relevant mutant reporter vectors, namely pGL3‐XIST‐Mut, pGL3‐SNHG5‐Mut and pGL3‐CARHSP1‐Mut were constructed. Subsequently, pGL3‐XIST‐Wt, pGL3‐SNHG5‐Wt, pGL3‐XIST‐Mut, pGL3‐SNHG5‐Mut, pGL3‐CARHSP1‐Wt and pGL3‐CARHSP1‐Mut plasmids were cotransfected with miR‐155 mimic or miR‐NC into HCAECs. Forty‐eight hours later, firefly luciferase activity and Renilla luciferase activity of the cells were detected utilizing luciferase assay kit (Promega, USA), and the luciferase activity of samples was referred as the ratio of Renilla activity and firefly activity. All these experiments were repeated for ≥ 3 times.

### 2.10 RNA immunoprecipitation (RIP) assay

HCAECs were disintegrated by RIP lysate following instructions of EZ‐Magna RIP kit (Millipore, USA), and the products were centrifuged at 12 000 r/min for 10 minutes to collect supernatants. Subsequently, IgG antibody or Ago2 antibody was supplemented into magnetic bead–containing EP tubes which were added by 900 μL RIP buffer, including RNase inhibitor, proteinase inhibitor and DNase, and 100 μL supernatant of HCAEC in advance. After incubation at 4°C for overnight, the mixture was centrifuged at the speed of 12 000 r/min for 10 minutes, and supernatants were discarded. Afterwards, the samples were rinsed by 500 μL RIP wash buffer, and RNAs were purified by 15 μL DEPC and conserved at −80°C. HCAECs with addition of IgG antibody and Ago2 antibody were separately included into anti‐IgG group and anti‐Ago2 group, while HCAECs without any treatment were set as positive control group (ie Input group).

### 2.11 Statistical analyses

All the statistical analyses were performed using SPSS 16.0 software. Measurement data in the form of mean ± standard deviation (SD) were analysed by Student's *t* test and one‐way analysis of variance (ANOVA). On the other hand, the count data (n or %) were analysed by chi‐square test. And *P* < .05 was considered as statistically significant.

## 3 RESULTS

### 3.1 Correlation between XIST/SNHG5/miR‐155 expressions and clinical characteristics of AS patients

Comparing the baseline characteristics of 367 AS patients with 219 healthy controls, there revealed a higher proportion of smokers among the AS patients than among the healthy crowd (*P* = .003). More than that, the AS patients were associated with higher levels of triglyceride (TG), total cholesterol (TC), low‐density lipoprotein cholesterol (LDL‐C) and high‐sensitivity C‐reactive protein (hs‐CRP) than healthy controls (*P* < .05) (Table 1). However, hardly any statistical differences were observable between the two groups, with respect to mean age, gender ratio and drinking status (*P* > .05).

**Table 1:** Comparison of clinical features between atherosclerosis patients and healthy controls

Clinical features	Patients group	Control group	t/χ^2^	*P* value
Number	367	219		
Age	62.48 ± 8.53	60.29 ± 9.41	1.57	.117
Gender				
Female	142	97		
Male	225	122	1.78	.182
Degree				
1%‐25% stenosis	196			
26%‐50% stenosis	110			
>50% stenosis	61			
Smoking				
Yes	246	120		
No	121	99	8.76	.003
Alcohol				
Yes	221	115		
No	146	104	3.33	.068
TG (mmol/L)	1.91 ± 1.03	1.67 ± 0.58	3.16	.002
TC (mmol/L)	4.72 ± 1.16	4.41 ± 1.03	3.26	.001
HDL‐C (mmol/L)	1.09 ± 0.29	1.14 ± 0.37	1.82	.070
LDL‐C (mmol/L)	2.52 ± 0.73	2.31 ± 0.62	3.56	<.001
hs‐CRP	5.19 ± 2.31	3.48 ± 1.64	9.60	<.001

Abbreviations: hs‐CRP, high‐sensitivity C‐reactive protein; LDL‐C, low‐density lipoprotein cholesterol; TC, total cholesterol; TG, triglyceride.

**Figure 1:**
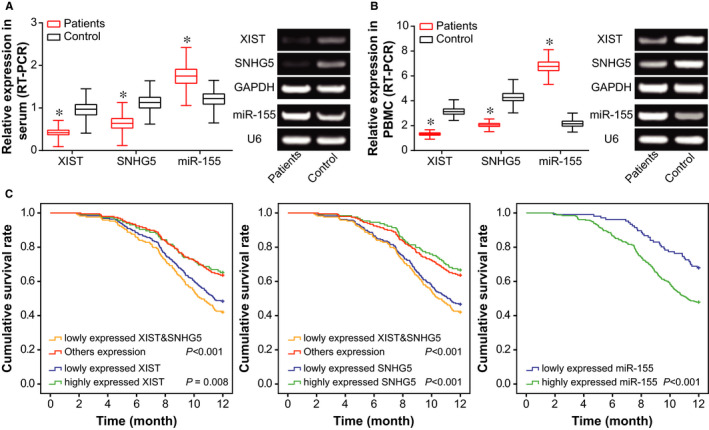
Relevance of XIST/SNHG5/miR‐155 expressions to clinical characteristics of AS patients. A, Serum levels of lncRNA XIST, lncRNA SNHG5 and miR‐155 were compared between AS patients and healthy controls. *: *P* < .05 when compared with healthy controls. B, Expressions of lncRNA XIST, lncRNA SNHG5 and miR‐155 within peripheral mononuclear cells were compared between AS patients and healthy controls. *: *P* < .05 when compared with healthy controls. C, Lowly expressed XIST/SNHG5 and highly expressed miR‐155 were positively relevant to the poor prognosis of AS patients

In addition, XIST and SNHG5 were expressed lowly within serum of AS patients in comparison to that of control group, yet miR‐155 expression exhibited a trend that was opposite to XIST and SNHG5 (*P* < .05) (Figure 1A). Levels of XIST, SNHG5 and miR‐155 within peripheral blood mononuclear cells (PBMCs) of AS patients and healthy controls assumed a tendency similar to their serum amount (*P* < .05) (Figure 1B). Moreover, with the mean serum level of XIST, SNHG5 and miR‐155, respectively, set as the cut‐off value, the AS population were separately divided into highly expressed (n = 115) and lowly expressed XIST group (n = 252), highly expressed (n = 129) and lowly expressed SNHG5 group (n = 238), as well as highly expressed (n = 261) and lowly expressed miR‐155 group (n = 106). Results in Table 2 indicated that lowly expressed XIST/SNHG5 and highly expressed miR‐155 were abundantly detectable among AS patients who demonstrated severe stenosis and possessed high levels of TG, LDL‐C and hs‐CRP, as compared with highly expressed XIST/SNHG5 and lowly expressed miR‐155 (*P* < .05). In the meantime, the Kaplan‐Meier curve clarified that AS patients with low XIST/SNHG5 expression and high miR‐155 expression exhibited shorter survival than those carrying high XIST/SNHG5 expression and low miR‐155 expression (*P* < .05) (Figure 1C). In addition, the AS patients who displayed low expression of both XIST and SNHG5 enjoyed shorter survival than those who did not present this trait (*P* < .05). Beyond that, the multivariate analyses insinuated that lowly expressed XIST/SNHG5, highly expressed miR‐155, severe stenosis and large amounts of TG/LDL‐C/hs‐CRP were independent elements for predicting unfavourable prognosis of AS patients (*P* < .05) (Table 3).

**Table 2:** Correlation between clinical features and lncRNA XIST/lncRNA SNHG5/miR‐486‐5p expression of atherosclerosis patients

Clinical features	LncRNA XIST expression				SNHG5 expression				miR‐155 expression			
	Low	High	χ^2^	*P* value	Low	High	χ^2^	*P* value	Low	High	χ^2^	*P* value
Age												
>62.48	153	79			157	75			62	170		
≤62.48	99	36	2.16	.141	81	54	2.20	.138	44	91	1.43	.232
Gender												
Female	94	48			88	54			45	97		
Male	158	67	0.66	.418	150	75	0.84	.359	61	164	0.89	.346
Degree												
1%‐25% stenosis	125	71			116	80			68	128		
26%‐50% stenosis	77	33			76	34			28	82		
>50% stenosis	50	11	7.29	.026	46	15	6.61	.037	10	51	8.48	.014
Smoking												
Yes	174	72			166	80			66	180		
No	78	43	1.48	.224	72	49	2.26	.133	40	81	1.53	.216
Alcohol												
Yes	158	63			149	72			59	162		
No	94	52	2.07	.151	89	57	1.61	.204	47	99	1.29	.256
TG (mmol/L)												
>1.91	177	67			167	77			62	182		
≤1.91	75	48	5.08	.024	71	52	4.12	.042	44	79	4.28	.039
TC (mmol/L)												
>4.72	164	65			158	71			60	169		
≤4.72	88	50	2.47	.116	80	58	4.59	.032	46	92	2.13	.144
HDL‐C (mmol/L)												
>1.09	96	55			91	60			53	98		
≤1.09	156	60	3.09	.079	147	69	2.37	.124	53	163	4.83	.028
LDL‐C (mmol/L)												
>2.52	181	70			173	78			63	188		
≤2.52	71	45	4.39	.036	65	51	5.78	.016	43	73	5.53	.019
hs‐CRP												
>5.19	162	58			152	68			53	167		
≤5.19	90	57	6.31	.012	86	61	4.33	.037	53	94	6.14	.013

Abbreviations: hs‐CRP, high‐sensitivity C‐reactive protein; LDL‐C, low‐density lipoprotein cholesterol; TC, total cholesterol; TG, triglyceride.

**Table 3:** Correlation between clinical features and atherosclerosis patients’ overall survival

Clinical features	Univariate analysis			Multivariate analysis		
	Hazard Ratio	95% CI	*P* value	Hazard Ratio	95% CI	*P* value
LncRNA XIST expression						
Low vs High	2.00	1.27‐3.15	.003	1.68	1.01‐2.78	.045
LncRNA SNHG5 expression						
Low vs High	2.29	1.47‐3.57	<.001	1.95	1.20‐3.18	.007
miR‐155 expression						
Low vs High	0.43	0.27‐0.70	.001	0.50	0.30‐0.85	.010
Age (years)						
>62.48 vs ≤62.48	0.98	0.64‐1.50	.919	0.91	0.56‐1.46	.686
Gender						
Female vs Male	1.01	0.66‐1.54	.962	1.19	0.74‐1.91	.465
Degree						
1%‐25% stenosis vs 26%‐50% stenosis	0.50	0.31‐0.81	.004	0.57	0.34‐0.95	.030
1%‐25% stenosis vs >50% stenosis	0.33	0.18‐0.60	<.001	0.46	0.24‐0.89	.020
Smoking history						
Yes vs No	0.95	0.62‐1.48	.832	0.88	0.54‐1.44	.608
Alcohol history						
Yes vs No	0.94	0.62‐1.43	.769	0.83	0.52‐1.33	.448
TG (mmol/L)						
>1.91 vs ≤1.91	2.24	1.43‐3.52	<.001	1.89	1.16‐3.08	.011
TC (mmol/L)						
>4.72 vs ≤4.72	1.15	0.75‐1.75	.528	0.98	0.61‐1.57	.939
HDL‐C (mmol/L)						
>1.09 vs ≤1.09	1.00	0.66‐1.52	.991	1.26	0.79‐2.01	.336
LDL‐C (mmol/L)						
>2.52 vs ≤2.52	2.16	1.36‐3.41	.001	1.68	1.02‐2.78	.043
hs‐CRP						
>5.19 vs ≤5.19	2.22	1.44‐3.41	<.001	1.74	1.08‐2.82	.023

Abbreviations: hs‐CRP, high‐sensitivity C‐reactive protein; LDL‐C, low‐density lipoprotein cholesterol; TC, total cholesterol; TG, triglyceride.

### 3.2 ox‐LDL promoted inflammatory response and apoptosis of HCAECs

**Figure 2:**
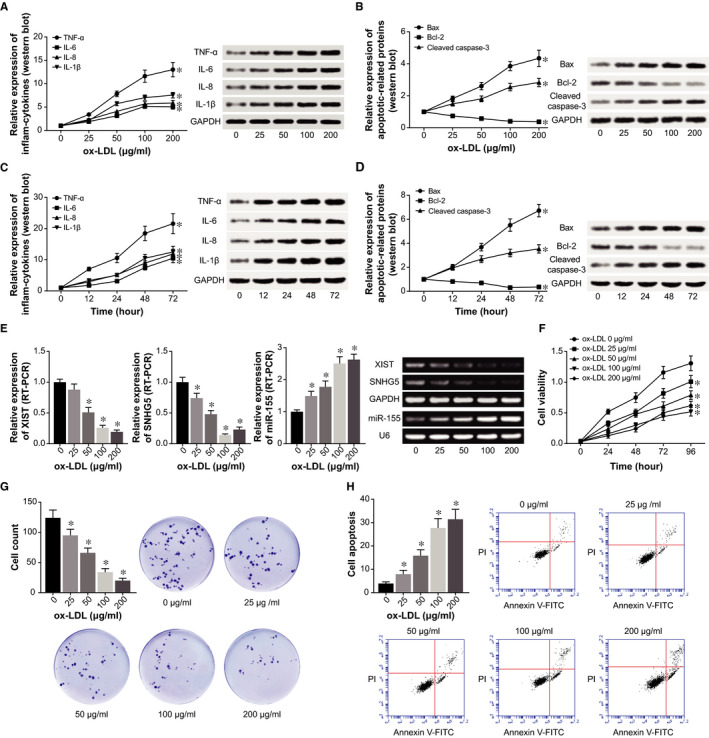
ox‐LDL promoted HCAECs’ release of inflammatory factors and also their apoptosis rate. Protein levels of (A) inflammatory cytokines (ie TNF‐α, IL‐6, IL‐8 and IL‐1β) and (B) apoptins (ie Bax, Bcl‐2 and cleaved caspase‐3) were quantified within HCAECs that were handled by ox‐LDL at concentrations of 0, 25, 50, 100 and 200 μg/m1 for 24 h (Western blot). *: *P* < .05 when compared with 0 μg/m1 group. Protein levels of (C) inflammatory cytokines, including TNF‐α, IL‐6, IL‐8 and IL‐1β, and (D) apoptins, including Bax, Bcl‐2 and cleaved caspase‐3, were measured within HCAECs that were treated by 100 μg/m1 ox‐LDL for 0, 12, 24, 48 and 72 h (Western blot). *: *P* < .05 when compared with 0 h. E, LncRNA XIST, lncRNA SNHG5 and miR‐155 expressions within HCAECs were monitored among treatments of 0, 25, 50, 100 and 200 μg/m1 ox‐LDL (RT‐PCR). *: *P* < .05 when compared with 0 μg/m1 group. F, Viability of HCAECs was contrasted when they were treated by 0, 25, 50, 100 and 200 μg/m1 ox‐LDL for 24 h. *: *P* < .05 when compared with 0 μg/m1 group. G, Colonies of HCAECs were counted when they were stimulated by 0, 25, 50, 100 and 200 μg/m1 ox‐LDL for 24 h. *: *P* < .05 when compared with 0 μg/m1 group. H, Apoptotic rates of HCAECs were calculated when they were managed by 0, 25, 50, 100 and 200 μg/m1 ox‐LDL for 24 h. *: *P* < .05 when compared with 0 μg/m1 group

The ox‐LDL of each concentration (ie 25, 50, 100 and 200 μg/mL) all could significantly raise the expressional level of inflammatory cytokines (ie TNF‐α, IL‐6, IL‐8 and IL‐1β) (Figure 2A) and apoptins (ie Bax, Bcl‐2 and cleaved caspase‐3) (Figure 2B) (all *P* < .05). However, this potentiation was abated when 100 μg/mL ox‐LDL was handled to treat the cells (*P* < .05). Besides, ox‐LDL (concentration: 100 μg/mL)‐induced expression of inflammatory factors (Figure 2C) and apoptins (Figure 2D) seemed to follow a time‐dependent manner. That was, the longer it took, the larger amount of inflammatory cytokines and apoptins produced by the HCAECs. Nonetheless, the variation degree became less pronounced at the time‐point of 72 hours (*P* < .05). Hence, ox‐LDL might enhance production of inflammatory and apoptotic biomarkers through time/quantity‐dependent fashions, and the most significant changes were observable when HCAECs were treated by 100 μg/m1 ox‐LDL for 48 hours.

Besides, with the incremental concentration of ox‐LDL, expressions of XIST and SNHG5 declined markedly in HCAECs, whereas miR‐155 expression took on a gradual ascent (*P* < .05) (Figure 2E). Additionally, driven by ox‐LDL, growth of the HCAECs was held back, while apoptosis of the HCAECs was promoted (*P* < .05) (Figure 2F‐H).

### 3.3 XIST cooperated with SNHG5 to encourage inflammatory response of HCAECs

**Figure 3:**
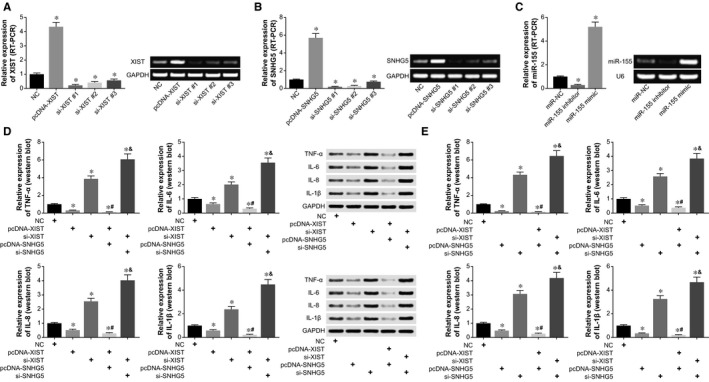
Impacts of XIST and SNHG5 on HCAECs’ release of inflammatory cytokines. A, XIST expression within HCAECs was determined after transfection of pcDNA‐XIST, si‐XIST #1, si‐XIST #2 and si‐XIST #3 (RT‐PCR). *: *P* < .05 when compared with NC group. B, SNHG5 expression was measured after transfecting HCAECs with pcDNA‐SNHG5, si‐SNHG5 #1, si‐SNHG5 #2 and si‐SNHG5 #3 (RT‐PCR). *: *P* < .05 when compared with NC group. C, Expression of miR‐155 within HCAECs was assessed after transfection of miR‐155 mimic and miR‐155 inhibitor (RT‐PCR). *: *P* < .05 when compared with NC group. D, Inflammatory cytokines produced by HCAECs were compared among NC, pcDNA‐XIST, si‐XIST, pcDNA‐XIST + pcDNA‐SNHG5 and si‐XIST + si‐SNHG5 groups (Western blot). *: *P* < .05 when compared with NC group; ^#^: *P* < .05 when compared with pcDNA‐XIST group; ^&^: *P* < .05 when compared with si‐XIST group. E, Inflammatory cytokines were determined within HCAECs that were treated by NC, pcDNA‐SNHG5, si‐SNHG5, pcDNA‐XIST + pcDNA‐SNHG5 and si‐XIST + si‐SNHG5 (Western blot). *: *P* < .05 when compared with NC group; ^#^: *P* < .05 when compared with pcDNA‐SNHG5 group; ^&^: *P* < .05 when compared with si‐SNHG5 group

The XIST expression of pcDNA‐XIST group was increased to 4.35‐fold more than that of control group (*P* < .05), while si‐XIST, on the contrary, evidently lowered XIST expression in the HCAECs (*P* < .05) (Figure 3A). Regarding SNHG5, its expression was overtly promoted by pcDNA‐SNHG5, yet was reduced markedly under the transfection of si‐SNHG5 #1, si‐SNHG5 #2 and si‐SNHG5 #3 (*P* < .05) (Figure 3B). Analogously, miR‐155 expression was prominently enhanced and inhibited in HCAECs that were treated by miR‐155 mimic and miR‐155 inhibitor, respectively (*P* < .05) (Figure 3C).

Based on the above treatments, we further discovered that either pcDNA‐XIST or pcDNA‐SNHG5 could evidently reduce the expression of TNF‐α, IL‐6, IL‐8 and IL‐1β, as relative to control group (*P* < .05) (Figure 3D,E). Co‐treatment of pcDNA‐XIST and pcDNA‐SNHG5 decreased expression levels of inflammatory cytokines more significantly than single treatment of pcDNA‐XIST or pcDNA‐SNHG5 (*P* < .05). By contrast, HCAECs of si‐XIST + si‐SNHG5 group produced higher levels of TNF‐α, IL‐6, IL‐8 and IL‐1β than those of si‐XIST group and si‐SNHG5 group (*P* < .05).

### 3.4 XIST in combination with SNHG5 influenced proliferation and apoptosis of HCAECs

**Figure 4:**
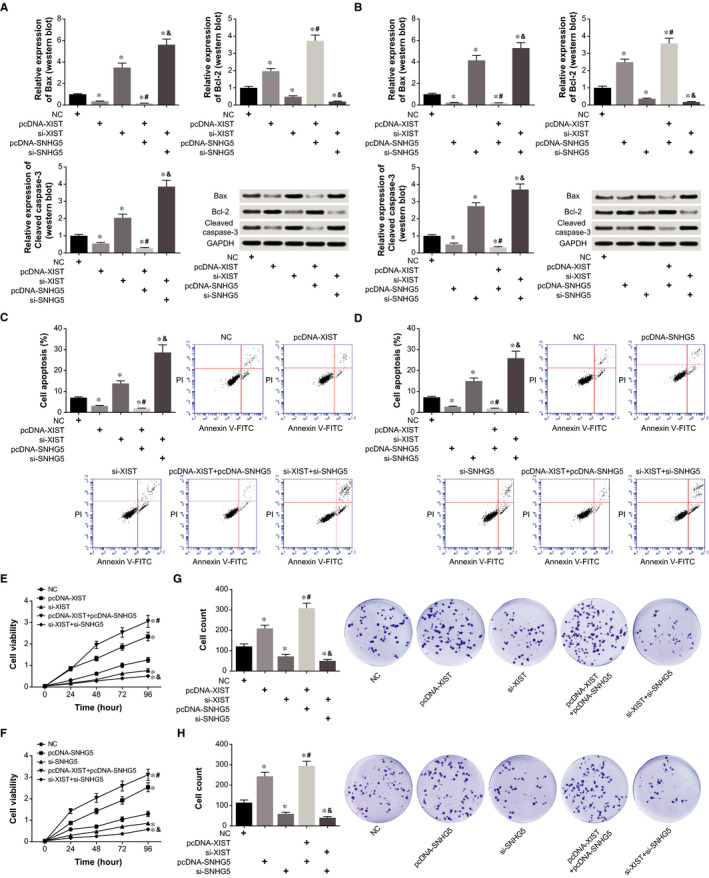
Combined influences of XIST and SNHG5 on proliferation and apoptosis of HCAECs. A, Expressions of apoptins were contrasted among HCAECs among NC, pcDNA‐XIST, si‐XIST, pcDNA‐XIST + pcDNA‐SNHG5 and si‐XIST + si‐SNHG5 groups (Western blot). *: *P* < .05 when compared with NC group; ^#^: *P* < .05 when compared with pcDNA‐XIST group; ^&^: *P* < .05 when compared with si‐XIST group. B, Apoptins expressed by HCAECs were determined among NC, pcDNA‐SNHG5, si‐SNHG5, pcDNA‐XIST + pcDNA‐SNHG5 and si‐XIST + si‐SNHG5 groups (Western blot). *: *P* < .05 when compared with NC group; ^#^: *P* < .05 when compared with pcDNA‐SNHG5 group; ^&^: *P* < .05 when compared with si‐SNHG5 group. C, The apoptotic rate of HCAECs was assessed when they were treated by NC, pcDNA‐XIST, si‐XIST, pcDNA‐XIST + pcDNA‐SNHG5 and si‐XIST + si‐SNHG5. *: *P* < .05 when compared with NC group; ^#^: *P* < .05 when compared with pcDNA‐XIST group; ^&^: *P* < .05 when compared with si‐XIST group. D, The apoptotic condition of HCAECs was appraised among NC, pcDNA‐SNHG5, si‐SNHG5, pcDNA‐XIST + pcDNA‐SNHG5 and si‐XIST + si‐SNHG5 groups. *: *P* < .05 when compared with NC group; ^#^: *P* < .05 when compared with pcDNA‐SNHG5 group; ^&^: *P* < .05 when compared with si‐SNHG5 group. E, Viability of HCAECs was analysed among NC, pcDNA‐XIST, si‐XIST, pcDNA‐XIST + pcDNA‐SNHG5 and si‐XIST + si‐SNHG5 groups. *: *P* < .05 when compared with NC group; ^#^: *P* < .05 when compared with pcDNA‐XIST group; ^&^: *P* < .05 when compared with si‐XIST group. F, HCAEC viability was compared among NC, pcDNA‐SNHG5, si‐SNHG5, pcDNA‐XIST + pcDNA‐SNHG5 and si‐XIST + si‐SNHG5 groups. *: *P* < .05 when compared with NC group; ^#^: *P* < .05 when compared with pcDNA‐SNHG5 group; ^&^: *P* < .05 when compared with si‐SNHG5 group. G, The proliferative capability of HCAECs was examined under transfections of NC, pcDNA‐XIST, si‐XIST, pcDNA‐XIST + pcDNA‐SNHG5 and si‐XIST + si‐SNHG5. *: *P* < .05 when compared with NC group; ^#^: *P* < .05 when compared with pcDNA‐XIST group; ^&^: *P* < .05 when compared with si‐XIST group. H, The multiplicative potential of HCAECs was judged among NC, pcDNA‐SNHG5, si‐SNHG5, pcDNA‐XIST + pcDNA‐SNHG5 and si‐XIST + si‐SNHG5 groups. *: *P* < .05 when compared with NC group; ^#^: *P* < .05 when compared with pcDNA‐SNHG5 group; ^&^: *P* < .05 when compared with si‐SNHG5 group

High expression of XIST and SNHG5 both lowered expressions of Bax and cleaved caspase‐3, and concurrently raised the expression of Bcl‐2 (*P* < .05) (Figure 4A,B). Moreover, expressions of the apoptins were altered more dramatically in the pcDNA‐XIST + pcDNA‐SNHG5 group than in the pcDNA‐XIST group and pcDNA‐SNHG5 group (*P* < .05). For another, expressions of Bax and cleaved caspase‐3 were promoted in both si‐XIST group and si‐SNHG5 group, when compared with control group (*P* < .05). Double transfection of si‐XIST and si‐SNHG5 led to lower Bcl‐2 expression and higher Bax and cleaved caspase‐3 expression than treatment of si‐XIST or si‐SNHG5 alone (*P* < .05). Furthermore, apoptosis of HCAECs exhibited a decline in the pcDNA‐XIST group and pcDNA‐SNHG5 group in comparison to NC group (*P* < .05), whereas low expression of XIST and SNHG5 both accelerated apoptosis of the HCAECs markedly (*P* < .05) (Figure 4C,D). On the contrary, viability and proliferative capacity of the HCAECs were largely reinforced by pcDNA‐XIST and pcDNA‐SNHG5 (*P* < .05), yet lowly expressed XIST and SNHG5 greatly weakened viability and proliferation of the HCAECs (*P* < .05) (Figure 4E‐H). In addition, co‐treatment of pcDNA‐XIST and pcDNA‐SNHG5 engendered more conspicuous impacts on viability and proliferation of HCAECs than pcDNA‐XIST/pcDNA‐SNHG5 alone, so did si‐XIST combined with si‐SNHG5 as relative to si‐XIST/si‐SNHG5 (*P* < .05).

### 3.5 XIST and SNHG5 sponged miR‐155 to down‐regulate its expression

**Figure 5:**
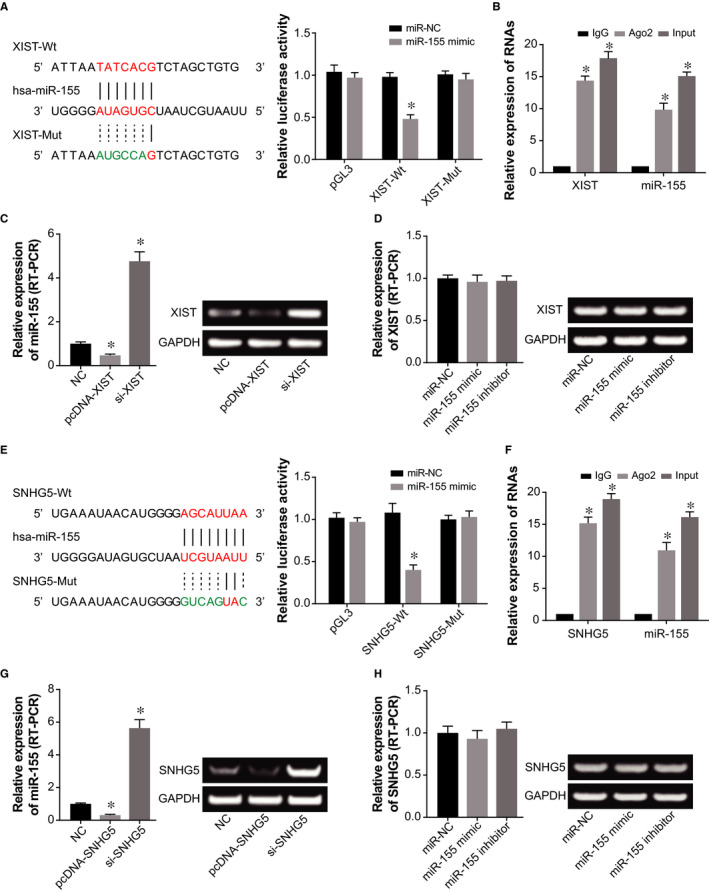
XIST and SNHG5 sponged and regulated miR‐155 in HCAECs. A, XIST sponged miR‐155, and the luciferase activities of HCAEC were compared among pGL3‐XIST‐Wt + miR‐155 mimic, pGL3‐XIST‐Mut + miR‐155 mimic and pGL3 + miR‐155 mimic groups. *: *P* < .05 when compared with pGL3‐XIST‐Wt + miR‐NC group. B, RIP results suggested that XIST and miR‐155 were concentrated in both anti‐Ago2 group and Input group. *: *P* < .05 when compared with anti‐lgG group. C, Expression of miR‐155 was influenced by pcDNA‐XIST and si‐XIST (RT‐PCR). *: *P* < .05 when compared with NC group. D, MiR‐155 mimic and miR‐155 inhibitor were appraised regarding their impacts on XIST expression (RT‐PCR). *: *P* < .05 when compared with NC group. E, MiR‐155 was sponged by SNHG5, and the luciferase activities of HCAEC were contrasted among pGL3‐SNHG5‐Wt + miR‐155 mimic, pGL3‐SNHG5‐Mut + miR‐155 mimic and pGL3 + miR‐155 mimic groups. *: *P* < .05 when compared with pGL3‐SNHG5‐Wt + miR‐NC group. F, RIP assay showed that SNHG5 and miR‐155 were enriched in anti‐Ago2 group and Input group. *: *P* < .05 when compared with anti‐lgG group. G, MiR‐155 expression was evaluated within HCAECs treated by pcDNA‐SNHG5 and si‐SNHG5 (RT‐PCR). *: *P* < .05 when compared with NC group. H, SNHG5 expression was detected within HCAECs after transfection of miR‐155 mimic and miR‐155 inhibitor (RT‐PCR). *: *P* < .05 when compared with NC group

The luciferase activity of HCAECs in the pGL3‐XIST‐Wt + miR‐155 mimic group was significantly decreased, as compared with pGL3‐XIST‐Mut + miR‐155 mimic group and pGL3 + miR‐155 mimic group (*P* < .05) (Figure 5A). Moreover, RIP results (Figure 5B) suggested that XIST and miR‐155 were concentrated in both anti‐Ago2 group and Input group as compared with anti‐lgG group (*P* < .05), and levels of XIST and miR‐155 were higher in Input group than in anti‐Ago2 group (*P* < .05). In addition, miR‐155 expression was restrained by overexpressed XIST and was elevated in case of under‐expressed XIST (*P* < .05) (Figure 5C). However, XIST expression revealed no significance difference under treatments of miR‐155 mimic and miR‐155 inhibitor (*P* > .05) (Figure 5D).

Similarly, pGL3‐SNHG5‐Wt cotransfected with miR‐155 mimic distinctly reduced luciferase activity of HCAECs, as relative to pGL3‐SNHG5‐Mut + miR‐155 mimic group and pGL3‐SNHG5‐Wt + miR‐NC group (*P* < .05) (Figure 5E). Both SNHG5 and miR‐155 were enriched in anti‐Ago2 group and Input group as relative to anti‐lgG group (*P* < .05), and the content of SNHG5 and miR‐155 in Input group was beyond that in anti‐Ago2 group (*P* < .05) (Figure 5F). Additionally, pcDNA‐SNHG5 treatment could lower miR‐155 expression, whereas miR‐155 expression was significantly boosted by si‐SNHG5 (*P* < .05) (Figure 5G). Nonetheless, hardly any changes of SNHG5 level were detectable within HCAECs of miR‐155 mimic group and miR‐155 inhibitor group, in comparison to NC group (*P* > .05) (Figure 5H). Summing up the above, there might be sponging relationships between XIST/SNHG5 and miR‐155, and miR‐155 expression was subjected to modulation of upstream XIST/SNHG5.

### 3.6 MiR‐155 hindered the contribution of XIST and SNHG5 to inflammatory response of HCAECs

**Figure 6:**
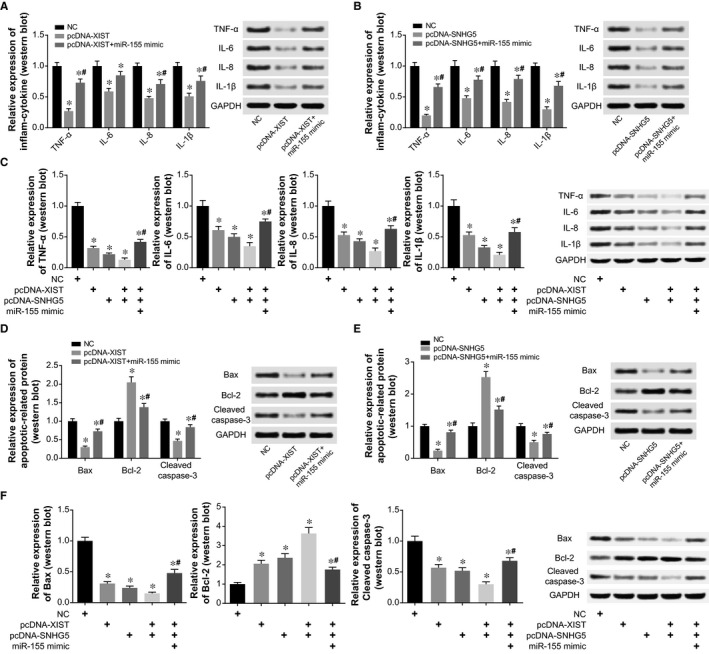
MiR‐155 mediated the effects of XIST and SNHG5 on the release of inflammatory factors and apoptins by HCAECs. A, Expressions of inflammatory cytokines were quantified within HCAECs handled by NC, pcDNA‐XIST and pcDNA‐XIST + miR‐155 mimic (Western blot). *: *P* < .05 when compared with NC group; ^#^: *P* < .05 when compared with pcDNA‐XIST group. B, Levels of inflammatory cytokines were determined within HCAECs under treatments of NC, pcDNA‐SNHG5 and pcDNA‐SNHG5 + miR‐155 mimic (Western blot). *: *P* < .05 when compared with NC group; ^#^: *P* < .05 when compared with pcDNA‐SNHG5 group. C, Expressions of inflammatory cytokines were drawn from HCAECs of NC, pcDNA‐XIST, pcDNA‐SNHG5, pcDNA‐XIST + pcDNA‐SNHG5 and pcDNA‐XIST + pcDNA‐SNHG5 + miR‐155 mimic groups (Western blot). *: *P* < .05 when compared with NC group; ^#^: *P* < .05 when compared with pcDNA‐XIST + pcDNA‐SNHG group. D, Expressions of apoptins were measured within HCAECs transfected by NC, pcDNA‐XIST and pcDNA‐XIST + miR‐155 mimic (Western blot). *: *P* < .05 when compared with NC group; ^#^: *P* < .05 when compared with pcDNA‐XIST group. E, Apoptin levels were monitored within HCAECs under treatments of NC, pcDNA‐SNHG5 and pcDNA‐SNHG5 + miR‐155 mimic (Western blot). *: *P* < .05 when compared with NC group; ^#^: *P* < .05 when compared with pcDNA‐SNHG5 group. F, Expressions of apoptins were contrasted among NC, pcDNA‐XIST, pcDNA‐SNHG5, pcDNA‐XIST + pcDNA‐SNHG5 and pcDNA‐XIST + pcDNA‐SNHG5 + miR‐155 mimic groups (Western blot). *: *P* < .05 when compared with NC group; ^#^: *P* < .05 when compared with pcDNA‐XIST + pcDNA‐SNHG group

Compared with NC group, highly expressed XIST/SNHG5 restrained expressions of TNF‐α, IL‐6, IL‐8 and IL‐1β notably, but this inhibitory role was alleviated when miR‐155 mimic was cotransfected (*P* < .05) (Figure 6A,B). To be specific, the expressional amount of inflammatory factors in the pcDNA‐XIST + miR‐155 mimic group and pcDNA‐SNHG5 + miR‐155 mimic group went up evidently, when compared with pcDNA‐XIST group and pcDNA‐SNHG5 group (*P* < .05). Furthermore, the expression of inflammatory cytokines in the pcDNA‐XIST + pcDNA‐SNHG5 + miR‐155 mimic group was above that in the pcDNA‐XIST + pcDNA‐SNHG5 group, reflecting that miR‐155 mimic could reverse the combined effects of pcDNA‐XIST and pcDNA‐SNHG5 (*P* < .05) (Figure 6C).

### 3.7 MiR‐155 intervened in the role of XIST and SHHG5 in modifying HCAEC apoptosis

**Figure 7:**
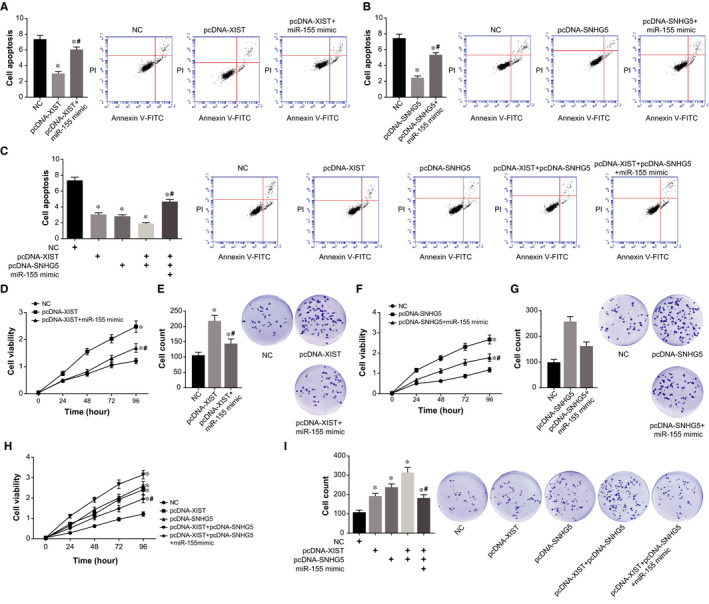
MiR‐155 intervened in the role of XIST and SHHG5 in modifying HCAEC apoptosis and proliferation. A, Apoptosis of HCAECs was evaluated among treatments of NC, pcDNA‐XIST and pcDNA‐XIST + miR‐155 mimic. *: *P* < .05 when compared with NC group; ^#^: *P* < .05 when compared with pcDNA‐XIST group. B, Apoptosis rate was calculated from HCAECs in NC, pcDNA‐SNHG5 and pcDNA‐SNHG5 + miR‐155 mimic groups. *: *P* < .05 when compared with NC group; ^#^: *P* < .05 when compared with pcDNA‐SNHG5 group. C, Apoptotic tendency of HCAECs was compared among NC, pcDNA‐XIST, pcDNA‐SNHG5, pcDNA‐XIST + pcDNA‐SNHG5 and pcDNA‐XIST + pcDNA‐SNHG5 + miR‐155 mimic groups. *: *P* < .05 when compared with NC group; ^#^: *P* < .05 when compared with pcDNA‐XIST + pcDNA‐SNHG group. D, Viability and (E) proliferative ability of HCAECs were determined among NC, pcDNA‐XIST and pcDNA‐XIST + miR‐155 mimic groups. *: *P* < .05 when compared with NC group; ^#^: *P* < .05 when compared with pcDNA‐XIST group. HCAECs treated by NC, pcDNA‐SNHG5 and pcDNA‐SNHG5 + miR‐155 mimic were assessed about their viability (F) and proliferation (G). *: *P* < .05 when compared with NC group; ^#^: *P* < .05 when compared with pcDNA‐SNHG5 group. Both viability (H) and proliferation (I) were determined among HCAECs managed by NC, pcDNA‐XIST, pcDNA‐SNHG5, pcDNA‐XIST + pcDNA‐SNHG5 and pcDNA‐XIST + pcDNA‐SNHG5 + miR‐155 mimic. *: *P* < .05 when compared with NC group; ^#^: *P* < .05 when compared with pcDNA‐XIST + pcDNA‐SNHG5 group

After transfection of pcDNA‐XIST or pcDNA‐SNHG5, expressions of Bax and cleaved caspase‐3 were decreased in HCAECs, yet Bcl‐2 expression was heightened (*P* < .05) (Figure 6D,E). However, highly expressed miR‐155 partly blocked the effects of pcDNA‐XIST and pcDNA‐SNHG5 on expression of apoptins in HCAECs (*P* < .05) (Figure 6F). Furthermore, apoptosis of HCAECs was prohibited by pcDNA‐XIST and pcDNA‐SNHG5, yet miR‐155 mimic could attenuate the hindering role exerted by pcDNA‐XIST and pcDNA‐SNHG5 on HCAEC apoptosis (*P* < .05) (Figure 7A‐C). On the other hand, up‐regulated XIST/SNHG5 expression intensified viability and proliferation of HCAECs to around twofold of NC group, yet additional treatment of miR‐155 mimic diminished this enhancement (*P* < .05) (Figure 7D‐G). The viability and proliferation of HCAECs in the pcDNA‐XIST + pcDNA‐XIST group were also weakened greatly by miR‐155 mimic (*P* < .05) (Figure 7H,I).

### 3.8 MiR‐155 targeted CARHSP1 to regulate inflammatory response and apoptosis of HCAECs

**Figure 8:**
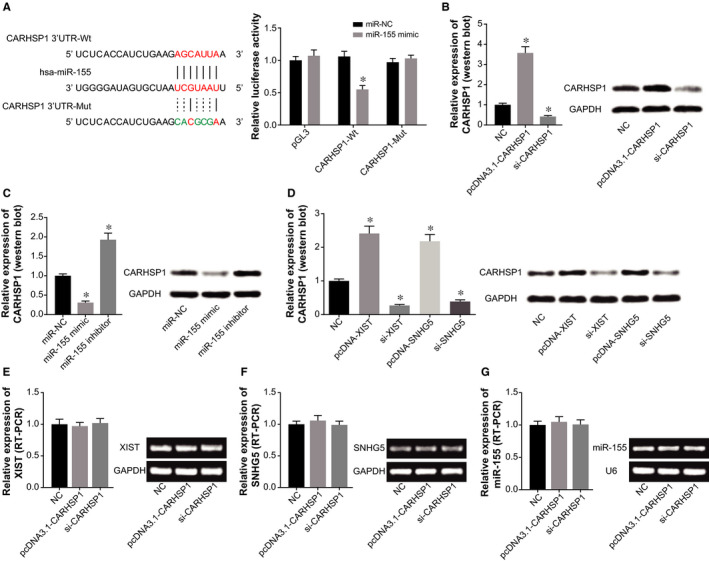
CARHSP1 expression was regulated by SNHG5, XIST and miR‐155. A, CARHSP1 was targeted by miR‐155 in certain sites, and luciferase activity of HCAECs was compared among pGL3‐CARHSP1‐Wt + miR‐155 mimic group, pGL3‐CARHSP1‐Mut + miR‐155 mimic group and pGL3 + miR‐155 mimic group.*: *P* < .05 when compared with pGL3 + miR‐155 mimic group. B, CARHSP1 expression was determined in HCAECs treated by NC, pcDNA3.1‐CARHSP1 and si‐CARHSP1. *: *P* < .05 when compared with NC group. C, CARHSP1 expression was measured among HCAECs in NC, miR‐155 mimic and miR‐155 inhibitor group. *: *P* < .05 when compared with NC group. D, CARHSP1 expression was detected among HCAECs of NC, pcDNA3.1‐XIST, si‐XIST, pcDNA3.1‐SNHG5 and si‐SNHG5 group. *: *P* < .05 when compared with NC group. (E‐G) XIST (E), SNHG5 (F) and miR‐155 (G) expressions in HCAECs were determined after transfections of none, pcDNA3.1‐CARHSP1 and si‐CARHSP1. *: *P* < .05 when compared with NC group

Luciferase activity of HCAECs in the pGL3‐CARHSP1‐Wt + miR‐155 mimic group was decreased than that in the pGL3‐CARHSP1‐Mut + miR‐155 mimic group and pGL3‐CARHSP1‐Mut + miR‐NC group (*P* < .05) (Figure 8A). Moreover, CARHSP1 expression in the pcDNA3.1‐CARHSP1 group was increased to 3.57 times of that in the pcDNA3.1 group, and CARHSP1 expression was reduced to 42% of NC group after silencing of CARHSP1 (*P* < .05) (Figure 8B). Furthermore, CARHSP1 expression in HCAECs was decreased by miR‐155 mimic, si‐XIST and si‐SNHG5 (*P* < .05), while miR‐155 inhibitor, pcDNA3.1‐XIST and pcDNA3.1‐SNHG5 significantly up‐regulated CARHSP1 expression (*P* < .05) (Figure 8C,D). However, expressions of miR‐155, XIST and SNHG5 were hardly changed when pcDNA3.1‐CARHSP1 and si‐CARHSP1 were transfected into HCAECs (*P* < .05) (Figure 8E‐G).

**Figure 9:**
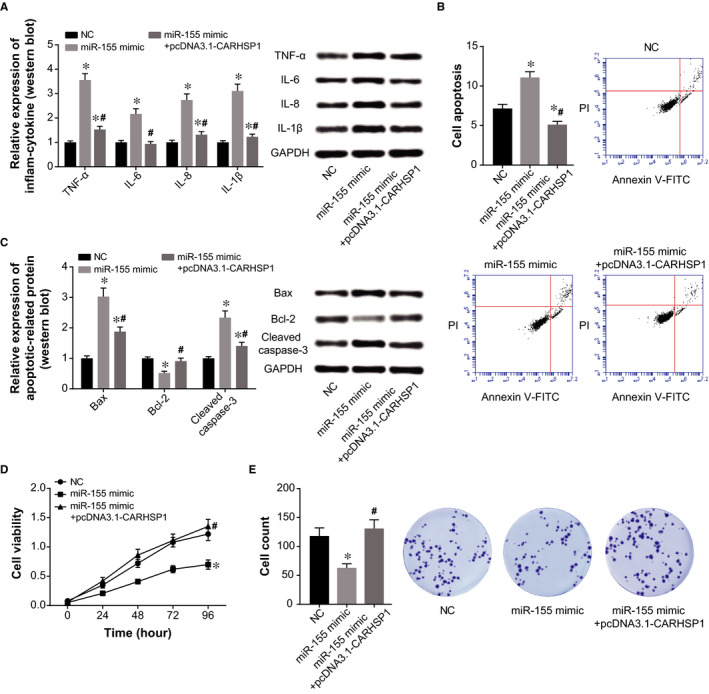
CARHSP1 was involved in the effect of miR‐155 on activity of HCAECs. A, Expressions of TNF‐α, IL‐6, IL‐8 and IL‐1β were determined in HCAECs of NC, miR‐155 mimic and miR‐155 mimic + pcDNA3.1‐CARHSP1 group. *: *P* < .05 when compared with NC group. B, Apoptotic percentage of HCAECs was determined after treatments of NC, miR‐155 mimic and miR‐155 mimic + pcDNA3.1‐CARHSP1. *: *P* < .05 when compared with NC group. C, Protein levels of Bax, Bcl‐2 and cleaved caspase‐3 were detected among HCAECs of NC, miR‐155 mimic and miR‐155 mimic + pcDNA3.1‐CARHSP1 group. *: *P* < .05 when compared with NC group. D‐E, Viability (D) and cell count (E) of HCAECs were assessed among NC, miR‐155 mimic and miR‐155 mimic + pcDNA3.1‐CARHSP1 group. *: *P* < .05 when compared with NC group

As illustrated in Figure 9A, protein levels of TNF‐α, IL‐6, IL‐8 and IL‐1β in HCAECs were down‐regulated by cotransfection of miR‐155 mimic and pcDNA3.1‐CARHSP1, when compared with miR‐155 mimic group (*P* < .05). Meanwhile, apoptosis of HCAECs was inhibited in the miR‐155 mimic + pcDNA3.1‐CARHSP1 group as relative to miR‐155 mimic group (*P* < .05) (Figure 9B). And cotransfection of miR‐155 mimic and pcDNA3.1‐CARHSP1 induced decreases of Bax/cleaved caspase‐3 expression and increases of Bcl‐2 expression in HCAECs, as compared with miR‐155 mimic group (*P* < .05) (Figure 9C). Furthermore, viability and proliferation of HCAECs were increased in the miR‐155 mimic + pcDNA3.1‐CARHSP1 group in comparison to miR‐155 mimic group (*P* < .05) (Figure 9D,E).

## 4 DISCUSSION

The onset of AS was partly acknowledged to result from endothelial dysfunction, specifically embodied as enhancive inflammatory cytokines released by impaired ECs.^18^ Notably, ox‐LDL was insinuated as a primary cause of vascular endothelial injury. In particular, the receptor structure of ox‐LDL was different from that of LDL, which made ox‐LDL no longer identifiable by macrophages. As a consequence, the ox‐LDL was readily devoured by macrophages, which led to incremental production of inflammatory cytokines (eg TNF‐α and IL‐8). For another, ox‐LDL could trigger apoptosis of ECs, which also motivated endothelial malfunction^19^ and thereby AS onset.^20^, ^21^, ^22^ Allowing for the considerable part of ox‐LDL underlying AS pathogenesis, here ox‐LDL was utilized to build cell models, and molecules that were involved in ox‐LDL‐induced AS onset were explored.

Robust evidences have highlighted the connection of lncRNAs with ox‐LDL accumulation underlying AS aetiology. For instance, expressional levels of lncRNAs MEG3,^23^ TUG1 ^24^ and MALAT1^25^ were raised by stimulation of ox‐LDL, and the lncRNAs were even capable of blocking ox‐LDL‐induced EC apoptosis. In the present investigation, we observed that lncRNAs XIST and SNHG5 became under‐expressed in ox‐LDL‐treated ECs (Figure 2E), suggesting their mediation of epithelial dysfunction underlying AS aetiology. More deeply, the XIST was revealed to strengthen proliferation of ECs (Figure 4E,G), and also restrain ox‐LDL‐induced inflammation in ECs (Figure 3D). Nonetheless, a contradiction was present between our results and the conclusion drawn by Han et al,^26^ which showed that XIST was a promoter of inflammation. Discrepancies of this sort could be ascribed to distinctions in the cell type, which demanded further experiments.

With regard to SNHG5, the mature spliceosome of U50,^27^ a majority of existing studies mainly emphasized its role in tumorigenesis, such as colorectal cancer, gastric cancer, breast cancer and melanoma.^15^, ^16^, ^28^ Nevertheless, finite researches were launched to correlate it with EC function. The current investigation might, to some extent, fill this gap, which demonstrated that overexpressed SNHG5 could restrain EC apoptosis (Figure 4D) and inflammation (Figure 3E). Actually, the apoptosis‐restraining role of SNHG5 was also detectable in certain tumours. For instance, overexpressed SNHG5 could antagonize oxaliplatin‐induced apoptosis of colorectal cancer cells,^15^ and elevating serum SNHG5 level was conjectured to urge metastasis of melanoma.^16^ Contrary to evidences as mentioned above, Zhao et al documented that SNHG5 was lowly expressed in gastric cancer, and its recruiting metastasis associated 1 family member 2 (MTA2) was involved in suppressing proliferation and metastasis of gastric cancer cells.^29^ It was thus conjectured that SNHG5 might function distinctly within various disorders, and specific accounts for SNHG5’ curbing EC apoptosis entailed thorough inquiries.

Taken together, it might be owing to these molecular mechanisms that serum levels of XIST and SNHG5 were clinically indicative of AS onset and prognosis (Figure 1C). There were implications that non‐coding RNAs in serum were possibly secreted by necrotic or apoptotic cells (eg HUVECs),^30^ or were derived from exosomes in blood.^31^ However, we failed to show how XIST and SNHG5 in HCAECs were released into serum of AS patients, because of shortfalls on technology. Huge efforts would be contributed to remedying this part in future. To further account for the role of XIST and SNHG5 in AS, here we proposed that the couple of lncRNAs might interfere with EC activity by sponging downstream miR‐155 (Figure 5A,E), just as revealed by the ceRNA theory.^32^ As a matter of fact, XIST and SNHG5 were previously documented to act upon miRNAs in other disorders. To be specific, XIST accelerated migration of human skin fibroblasts (HSFs) by sponging miR‐29a and restraining its expression.^33^ Besides, SNHG5 could sponge miR‐32 to elevate expression of Kruppel‐like factor 4 (KLF4), which altogether urged metastasis of gastric cancer cells.^28^ Herein it might be due to sponging miR‐155, as proved by dual luciferase reporter gene assay (Figure 5A,E) and RIP assay (Figure 5B,F), that XIST and SNHG5 were competent in reflecting AS development, on account of the multi‐dimensional function of miR‐155 in regulating EC function. For instance, miR‐155 expression within ECs was augmented by TNF‐α treatment, and concurrently stability of endothelial nitric oxide synthase was disturbed.^34^ Moreover, WEBER et al ^35^ documented that miR‐155 was likely to modulate RhoA and thereby reduce activity of eNOS,^36^, ^37^ which was a reflex of endothelial injury.^38^ In this study, CARHSP1 was newly introduced as the downstream molecule of miR‐155 in ECs (Figure 8A). The CARHSP1 was reported to enhance stability of TNF‐α mRNA, which could induce injury of vascular ECs,^39^ and depressing CARHSP1 expression could inhibit release of TNF‐α by foam cells in AS.^40^ Apart from the above, this investigation gained knowledge that CARHSP1 also mediated the role of miR‐155 in regulating inflammation and viability of HUVECs (Figure 9). In effect, the miR‐155 has been documented to regulate disease progression after being sponged by lncRNAs,^41^ which implied the persuasiveness of lncRNA‐miR‐155 axis in explaining AS onset. Back to XIST and SNHG5 investigated here, they were potentially associated with cytokine release and proliferation of ECs by sponging downstream miR‐155 (Figure 6A‐C) and promoting CARHSP1 expression (Figure 8D).

Above all, this investigation illuminated that targeting XIST and SNHG5 might be a desirable option in slowing down AS development, allowing for their negative regulation of miR‐155/CARHSP1 axis. Nonetheless, several points demanded improvements, and a series of studies should be performed to overcome them. Firstly, through which approach XIST, SNHG5 and miR‐155 were transported into serum should be explored, and if serum levels of XIST, SNHG5 and miR‐155 could reflect their expressional change in HUVECs also demanded in‐depth proofs. Secondly, other downstream genes of miR‐155 in modifying HUVEC dysfunction, other than CARHSP1, should also be tapped. Thirdly, mouse models of AS were not constructed here to estimate the role of XIST/SNHG5‐miR‐155‐CARHSP1 axis in AS pathogenesis. Results of this investigation might be more convincing if weaknesses mentioned above were remedied.

## CONFLICT OF INTEREST

None.

## AUTHOR CONTRIBUTION

**Xianjing Song:** Conceptualization (equal); Formal analysis (equal); Investigation (equal); Project administration (equal). **Chuang Yang:** Conceptualization (equal); Data curation (equal); Methodology (equal); Software (equal). **Jing Chang:** Conceptualization (equal); Funding acquisition (equal); Methodology (equal); Validation (equal); Visualization (equal). **Xin Xue:** Conceptualization (equal); Visualization (equal); Writing‐original draft (equal); Writing‐review & editing (equal).

## References

[1] Hansson GK . Inflammation, atherosclerosis, and coronary artery disease. New Engl J Med. 2005;352:1685‐1695. 15843671 10.1056/NEJMra043430

[2] Braunwald E . Shattuck lecture–cardiovascular medicine at the turn of the millennium: triumphs, concerns, and opportunities. New Engl J Med. 1997;337:1360‐1369. 9358131 10.1056/NEJM199711063371906

[3] Libby P , Bornfeldt KE , Tall AR . Atherosclerosis: successes, surprises, and future challenges. Circu Res. 2016;118:531‐534. 10.1161/CIRCRESAHA.116.308334 PMC4762065 26892955

[4] Nabel EG , Braunwald E . A tale of coronary artery disease and myocardial infarction. New Engl J Med. 2012;366:54‐63. 22216842 10.1056/NEJMra1112570

[5] Murdoch CE , Chaubey S , Zeng L , et al. Endothelial NADPH oxidase‐2 promotes interstitial cardiac fibrosis and diastolic dysfunction through proinflammatory effects and endothelial‐mesenchymal transition. J Am Coll Cardiol. 2014;63:2734‐2741. 24681145 10.1016/j.jacc.2014.02.572

[6] Zhu N , Zhang D , Chen S , et al. Endothelial enriched microRNAs regulate angiotensin II‐induced endothelial inflammation and migration. Atherosclerosis. 2011;215:286‐293. 21310411 10.1016/j.atherosclerosis.2010.12.024

[7] Pankratz F , Bemtgen X , Zeiser R , et al. MicroRNA‐155 exerts cell‐specific antiangiogenic but proarteriogenic effects during adaptive neovascularization. Circulation. 2015;131:1575‐1589. 25850724 10.1161/CIRCULATIONAHA.114.014579

[8] Tiong AY , Brieger D . Inflammation and coronary artery disease. Am Heart J. 2005;150:11‐18. 16084145 10.1016/j.ahj.2004.12.019

[9] Salmena L , Poliseno L , Tay Y , Kats L , Pandolfi PP . A ceRNA hypothesis: the Rosetta Stone of a hidden RNA language? Cell. 2011;146:353‐358. 21802130 10.1016/j.cell.2011.07.014 PMC3235919

[10] Lu W , Huang SY , Su L , Zhao BX , Miao JY . Long noncoding RNA LOC100129973 suppresses apoptosis by targeting miR‐4707‐5p and miR‐4767 in vascular endothelial cells. Sci Rep. 2016;6:21620. 26887505 10.1038/srep21620 PMC4757888

[11] He C , Ding JW , Li S , et al. The role of long intergenic noncoding RNA p21 in vascular endothelial cells. DNA Cell Biol. 2015;34:677‐683. 26273737 10.1089/dna.2015.2966

[12] Xu X , Ma C , Liu C , Duan Z , Zhang L . Knockdown of long noncoding RNA XIST alleviates oxidative low‐density lipoprotein‐mediated endothelial cells injury through modulation of miR‐320/NOD2 axis. Biochem Biophy Res Commun. 2018;503:586‐592. 10.1016/j.bbrc.2018.06.042 29902461

[13] Zheng R , Lin S , Guan L , et al. Long non‐coding RNA XIST inhibited breast cancer cell growth, migration, and invasion via miR‐155/CDX1 axis. Biochem Biophys Res Commun. 2018;498:1002‐1008. 29550489 10.1016/j.bbrc.2018.03.104

[14] Yan L , Wang S , Li Y , et al. SNHG5 promotes proliferation and induces apoptosis in melanoma by sponging miR‐155. RSC Adv. 2018;8:6160‐6168. 10.1039/c7ra12520h PMC9078272 35539582

[15] Damas ND , Marcatti M , Come C , et al. SNHG5 promotes colorectal cancer cell survival by counteracting STAU1‐mediated mRNA destabilization. Nat Commun. 2016;7:13875. 28004750 10.1038/ncomms13875 PMC5192221

[16] Ichigozaki Y , Fukushima S , Jinnin M , et al. Serum long non‐coding RNA, snoRNA host gene 5 level as a new tumor marker of malignant melanoma. Exp Dermatol. 2016;25:67‐69. 26440365 10.1111/exd.12868

[17] Sullivan DR , Marwick TH , Freedman SB . A new method of scoring coronary angiograms to reflect extent of coronary atherosclerosis and improve correlation with major risk factors. Am Heart J. 1990;119:1262‐1267. 1972310 10.1016/s0002-8703(05)80173-5

[18] Garcia de Tena J . Inflammation, atherosclerosis, and coronary artery disease. New Engl J Med. 2005;353:429‐430; author reply ‐30. 10.1056/NEJM200507283530425 16049220

[19] Dimmeler S , Zeiher AM . Endothelial cell apoptosis in angiogenesis and vessel regression. Circul Res. 2000;87:434‐439. 10.1161/01.res.87.6.434 10988233

[20] Harada‐Shiba M , Kinoshita M , Kamido H , Shimokado K . Oxidized low density lipoprotein induces apoptosis in cultured human umbilical vein endothelial cells by common and unique mechanisms. J Biol Chem. 1998;273:9681‐9687. 9545302 10.1074/jbc.273.16.9681

[21] Bombeli T , Karsan A , Tait JF , Harlan JM . Apoptotic vascular endothelial cells become procoagulant. Blood. 1997;89:2429‐2442. 9116287

[22] Bombeli T , Schwartz BR , Harlan JM . Endothelial cells undergoing apoptosis become proadhesive for nonactivated platelets. Blood. 1999;93:3831‐3838. 10339490

[23] Zhang Y , Liu X , Bai X , et al. Melatonin prevents endothelial cell pyroptosis via regulation of long noncoding RNA MEG3/miR‐223/NLRP3 axis. J Pineal Res. 2018;64:e12449. 10.1111/jpi.12449 29024030

[24] Chen C , Cheng G , Yang X , Li C , Shi R , Zhao N . Tanshinol suppresses endothelial cells apoptosis in mice with atherosclerosis via lncRNA TUG1 up‐regulating the expression of miR‐26a. Am J Transl Res. 2016;8:2981‐2991. 27508018 PMC4969434

[25] Tang Y , Jin X , Xiang Y , et al. The lncRNA MALAT1 protects the endothelium against ox‐LDL‐induced dysfunction via upregulating the expression of the miR‐22‐3p target genes CXCR2 and AKT. FEBS Lett. 2015;589:3189‐3196. 26364720 10.1016/j.febslet.2015.08.046

[26] Han Y , Ma J , Wang J , Wang L . Silencing of H19 inhibits the adipogenesis and inflammation response in ox‐LDL‐treated Raw264.7 cells by up‐regulating miR‐130b. Mol Immunol. 2018;93:107‐114. 29172088 10.1016/j.molimm.2017.11.017

[27] Pacilli A , Ceccarelli C , Trere D , Montanaro L . SnoRNA U50 levels are regulated by cell proliferation and rRNA transcription. Int J Mol Sci. 2013;14:14923‐14935. 23867608 10.3390/ijms140714923 PMC3742280

[28] Zhao L , Han T , Li Y , et al. The lncRNA SNHG5/miR‐32 axis regulates gastric cancer cell proliferation and migration by targeting KLF4. FASEB J. 2017;31:893‐903. 27871067 10.1096/fj.201600994R

[29] Zhao L , Guo H , Zhou B , et al. Long non‐coding RNA SNHG5 suppresses gastric cancer progression by trapping MTA2 in the cytosol. Oncogene. 2016;35:5770‐5780. 27065326 10.1038/onc.2016.110

[30] Ren S , Wang F , Shen J , et al. Long non‐coding RNA metastasis associated in lung adenocarcinoma transcript 1 derived miniRNA as a novel plasma‐based biomarker for diagnosing prostate cancer. Eur J Cancer. 2013;49:2949‐2959. 23726266 10.1016/j.ejca.2013.04.026

[31] Li Q , Shao Y , Zhang X , et al. Plasma long noncoding RNA protected by exosomes as a potential stable biomarker for gastric cancer. Tum Biol. 2015;36:2007‐2012. 10.1007/s13277-014-2807-y 25391424

[32] Song X , Cao G , Jing L , et al. Analysing the relationship between lncRNA and protein‐coding gene and the role of lncRNA as ceRNA in pulmonary fibrosis. J Cell Mol Med. 2014;18:991‐1003. 24702795 10.1111/jcmm.12243 PMC4508140

[33] Guo L , Huang X , Liang P , et al. Role of XIST/miR‐29a/LIN28A pathway in denatured dermis and human skin fibroblasts (HSFs) after thermal injury. J Cell Biochem. 2018;119:1463‐1474. 28771809 10.1002/jcb.26307

[34] Sun HX , Zeng DY , Li RT , et al. Essential role of microRNA‐155 in regulating endothelium‐dependent vasorelaxation by targeting endothelial nitric oxide synthase. Hypertension. 2012;60:1407‐1414. 23108656 10.1161/HYPERTENSIONAHA.112.197301

[35] Weber M , Kim S , Patterson N , Rooney K , Searles CD . MiRNA‐155 targets myosin light chain kinase and modulates actin cytoskeleton organization in endothelial cells. Am J Physiol Heart Circul Physiol. 2014;306:H1192‐H1203. 10.1152/ajpheart.00521.2013 PMC3989748 24486510

[36] Arita R , Nakao S , Kita T , et al. A key role for ROCK in TNF‐alpha‐mediated diabetic microvascular damage. Invest Ophthalmol Vis Sci. 2013;54:2373‐2383. 23462755 10.1167/iovs.12-10757

[37] Wolfrum S , Dendorfer A , Rikitake Y , et al. Inhibition of Rho‐kinase leads to rapid activation of phosphatidylinositol 3‐kinase/protein kinase AKT and cardiovascular protection. Arterioscl, Thromb Vasc Biol. 2004;24:1842‐1847. 15319269 10.1161/01.ATV.0000142813.33538.82 PMC2649731

[38] Zhou Q , Gensch C , Liao JK . Rho‐associated coiled‐coil‐forming kinases (ROCKs): potential targets for the treatment of atherosclerosis and vascular disease. Trends Pharmacol Sci. 2011;32:167‐173. 21242007 10.1016/j.tips.2010.12.006 PMC3080120

[39] Qin B , Shu Y , Xiao L , et al. MicroRNA‐150 targets ELK1 and modulates the apoptosis induced by ox‐LDL in endothelial cells. Mol Cell Biochem. 2017;429:45‐58. 28110404 10.1007/s11010-016-2935-3

[40] Jang SY , Yoon JS . Role of miR‐146a in the Regulation of Inflammation in an In Vitro Model of Graves' Orbitopathy. Invest Ophthalmol Vis Sci. 2016;57:6796. 28002568 10.1167/iovs.16-20847

[41] Li S , Sun Y , Zhong L , et al. The suppression of ox‐LDL‐induced inflammatory cytokine release and apoptosis of HCAECs by long non‐coding RNA‐MALAT1 via regulating microRNA‐155/SOCS1 pathway. Nutr Metab Cardiovascu. 2018;28:1175‐1187. 10.1016/j.numecd.2018.06.017 30314869

